# *In situ* self-assembly of Au-antimiR-155 nanocomplexes mediates TLR3-dependent apoptosis in hepatocellular carcinoma cells

**DOI:** 10.18632/aging.103799

**Published:** 2020-11-05

**Authors:** Liang Yin, Weijuan Cai, Yongqian Liang, Jie Yao, Xuemei Wang, Jie Shen

**Affiliations:** 1 Department of Endocrinology, Shunde Hospital of Southern Medical University, The First People's Hospital of Shunde Foshan, Shunde 528300, P. R. China; 2 State Key Laboratory of Bioelectronics, Chien-Shiung Wu Lab, School of Biological Science and Medical Engineering, Southeast University, Nanjing 210096, P. R. China; 3 Central Laboratory, Shunde Hospital of Southern Medical University, The First People's Hospital of Shunde Foshan, Shunde 528300, P. R. China

**Keywords:** Au-antimiR-155 NCs, Toll-like receptor 3, NF-κB, Caspase-8, hepatocellular carcinoma

## Abstract

MicroRNA 155 (miRNA-155) is frequently dysregulated in hepatocellular carcinoma (HCC) and other cancer types. Toll-like receptor 3 (TLR3), a putative miR-155 target, plays a key role in liver pathophysiology, and its downregulation in HCC cells is associated with apoptosis evasion and poor outcomes. Herein, we examined the ability of *in situ* self-assembled Au-antimiR-155 nanocomplexes (Au-antimiRNA NCs) to activate TLR3 signaling in HCC cells. Gene expression analysis confirmed an inverse relationship between miR-155 and TLR3 expression in HCC samples, and marked upregulation of miR-155 was observed in HCC cells but not in normal L02 hepatocytes. RNA immunoprecipitation confirmed physical interaction between miR-155 and TLR3, while negative regulation of TLR3 expression by miR-155 was demonstrated by luciferase reporter assays. Au-antimiR-155 NCs were self-assembled within HepG2 HCC cells, but not within control L02 cells. They efficiently silenced miR-155, thereby inhibiting proliferation and migration and inducing apoptosis in HepG2 cells. Molecular analyses suggested these effects are secondary to TLR3 signaling mediating NF-κB transcription, caspase-8 activation, and interleukin-1β (IL-1β) release. Our results provide a basis for future studies examining the *in vivo* applicability of this novel Au-antimiRNA NCs delivery system to halt HCC progression by activating pro-apoptotic TLR3 signaling.

## INTRODUCTION

Hepatocellular carcinoma (HCC) is the fifth most common cancer and the third leading cause of cancer-related deaths in the world [1]. Due to the difficulty of early diagnosis, HCC is commonly detected at advanced stages, resulting in a 5-year survival rate of only 15%-38%. The distribution of population risk factors for HCC is highly variable and may change according to the geographic region examined and to racial or ethnic group composition [2]. Xinjiang is a multi-ethnic settlement region in northwestern China. Xinjiang’s ethnic Kazakhs have traditional distinctive living habits and dietary structures. In turn, research suggested that distinct pathogenic features characterize HCC development or progression in Kazakh people and other minorities in the Xinjiang region [3]. The incidence of HCC in Xinjiang’s Kazakhs ranks sixth among all tumors and third among malignancies of the digestive tract. Despite being considered a population at high-risk for HCC [4], the pathogenic mechanisms of HCC within this demographic group have not yet been elucidated.

MicroRNAs (miRNAs) are a class of non-coding RNAs, about 20-25 nucleotides in length, that affect the stability and translation of genes by binding to the 3’-untranslated region (UTR) of target mRNAs. A complex regulatory network conformed by miRNA and target genes is involved in various physiological and pathological processes such as cell growth, differentiation, inflammation, and cancer. Aberrant miRNA expression contributes to the development and progression of many cancer types, including HCC [5–8]. Studies have shown that miR-155 is one of the most commonly upregulated miRNAs in tumors [9]. For instance, altered miR-155 expression has been reported in glioma [10], liver [11] and esophageal cancer [12]. Research has shown that miR-155 also contributes to the pathogenesis of HCC and suggested that miR-155-targeting therapies may be relevant for HCC treatment [13].

Toll-like receptors (TLRs) belong to the pattern recognition receptor (PRR) family that include proteins that recognize pathogen-associated molecular patterns (PAMPs) [14]. Among the TLRs, TLR3, 7, 8, and 9 recognize viral or bacterial nucleic acids. Either viral or host-derived double-stranded RNA serves as the molecular pattern recognized by TLR3 [15]. During chronic liver infection by hepatitis B virus (HBV), impaired TLR3 expression and onset of immunosuppressive cellular responses may be fundamental for the progression to cirrhosis and HCC [16]. Indeed, past studies suggested that TLR3 plays a key role in liver pathophysiology and its downregulation in HCC cells is associated with apoptosis evasion and poor prognosis [17, 18]. NF-κB activation and production of cytokines such as IL-1β mediate typical pro-inflammatory responses triggered by TLRs pathways [19]. However, the apoptotic pathway is preferentially induced over the pro-inflammatory pathway upon TLR3 activation in HCC cells [20]. Recent research demonstrated extensive cross-regulation between miRNAs and TLRs and identified miR-155 as a putative TLR3 regulator [21–23]. Therefore, TLR3 activation may be a novel and powerful strategy for the treatment of HBV-mediated chronic hepatitis B infections and HCC [24].

MiRNA inhibitors (antimiRNA) are chemically modified oligonucleotides designed to target specific miRNAs [25]. The introduction of miRNA inhibitors into cells allows to conduct loss-of-function assays to test regulatory interactions between endogenous miRNA/mRNA duplexes. Synthetic inhibitors are generally a non-hydrolyzable, single-strand reverse complement to the mature miRNA [25]. Novel approaches to the chemical synthesis of miRNA inhibitors have been pursued in recent years to increase biocompatibility, specificity, and therapeutic efficacy in cancer and other pathologies [26–27]. In this work, we examine the co-expression pattern of miR-155 and TLR3 in HCC and present a cell-based approach for *in situ* generation of self-assembled Au-antimiRNA nanocomplexes (Au-antimiRNA NCs) for use in miRNA inhibitor delivery systems. In addition, we assessed the potential of this nanotherapeutic system by testing the ability of Au-antimiR-155 NCs to upregulate TLR3 expression and promote apoptosis in cultured HCC cells.

## RESULTS

### MiR-155 is highly expressed in human HCC tissues and cell lines

**Figure 1:**
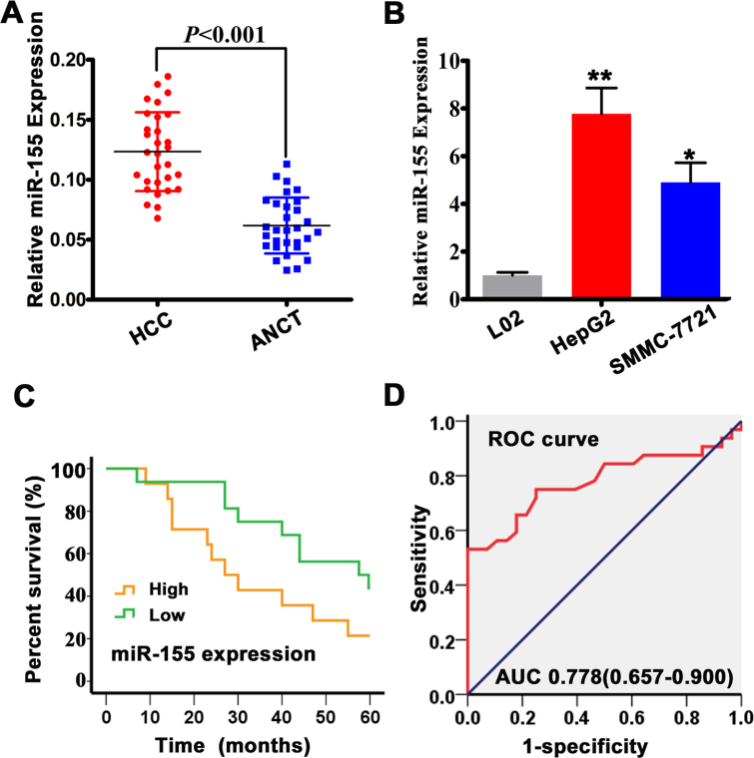
**MiR-155 is overexpressed in HCC tissues and cells.** (**A**) miR-155 expression in HCC and adjacent non-cancer tissues (ANCT) was measured by qRT-PCR (n=30; *P*<0.05 vs. ANCT). (**B**) qRT-PCR analysis of miR-155 transcripts in L02, HepG2, and SMMC-7721 cells (n=3; **P<0.01, *P<0.05; HepG2/SMMC-7721 vs L02 cells). (**C**) Kaplan-Meier analysis showing the association between miR-155 expression and overall survival (OS) in HCC patients. (**D**) ROC curve and AUC statistics indicating the diagnostic performance of high miR-155 expression on HCC. Error bars represent the SD of the mean from at least three independent experiments.

To verify the expression of miR-155 in HCC, we interrogated a panel of 30 human HCC specimens and corresponding adjacent non-cancer tissues (ANCT) by qRT-PCR. Our results confirmed overall overexpression of miR-155 in HCC samples (Figure 1A). As shown in Figure 1B the expression of miR-155 was also increased in human hepatocellular carcinoma cell lines (HepG2 and SMMC-7721) compared to normal embryonic hepatocytes (L02). Therefore, HepG2 cells were selected for subsequent *in vitro* studies. To determine the prognostic impact of miR-155 expression in HCC, we categorized HCC patients into two groups (i.e. high and low miR-155 expression) relative to ANCT. Overall survival (OS) was calculated as the time from diagnosis to the date of death or last contact and was used to evaluate the association between miR-155 expression levels and prognosis in HCC patients. High miR-155 expression levels were associated with significantly shorter patient survival compared with low miR-155 expression (Figure 1C). In addition, receiver operating characteristic (ROC) curves were plotted to test the diagnostic value of high miR-155 expression on HCC (Figure 1D). The results showed good diagnostic performance, evidenced by an area under the curve (AUC) of 0.778 (95% CI: 0.657-0.900).

### Evaluation of *in situ* self-assembly of Au-antimiR-155 NCs

**Figure 2:**
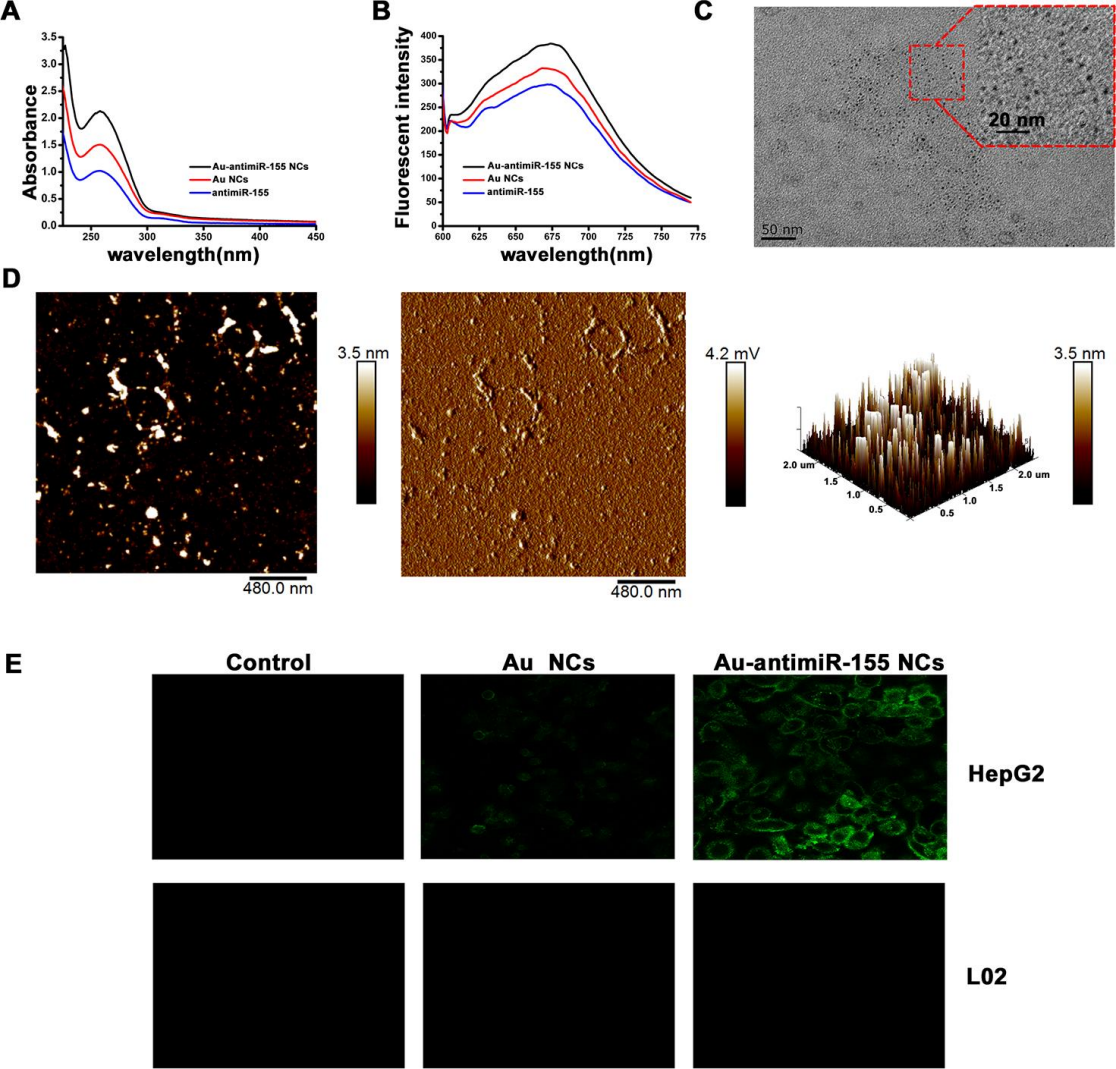
**Characterization of *in situ* assembled fluorescent Au-antimiR-155 NCs.** (**A**) UV absorption spectra of Au-antimiR-155 complexes extracted from HepG2 cells cultured with gold precursor and miR-155 inhibitors. (**B**) Fluorescence spectra of the aqueous solution of the extracted Au-antimiR-155 complexes. The relevant emission peak is centered at ~680 nm after excitation (EX) at 580 nm. (**C**) Representative TEM image of the isolated Au-antimiR-155 NCs. (**D**) Representative AFM image (left panel) and 3D model diagram (right panel) of biosynthetic Au-antimiR-155 NCs. The AFM phase diagram shown in the middle panel allows distinction of antimiR-155 and Au-antimiR-155 NCs. (**E**) Laser confocal fluorescence images of control (DMEM) cells and cells cultured in the presence of gold precursor alone (Au NCs) or gold precursor plus antimiR-155 (Au-antimiR-155 NCs). In the above experiments, the concentration of gold precursor solution was 5 μM and the concentration of antimiR-155 was 100 nM.

To characterize the *in situ* formation of chemically modified antimiR-155 oligonucleotides, we treated HepG2 cells with chloroauric acid (HAuCl_4_) and antimiR-155 oligonucleotides. Using extracts obtained from proliferating cells, *in situ* formation of Au-antimiRNA NCs was evaluated by UV-Vis spectrometry. Consistent with previous data [28], results showed a main absorption peak in the range 250-300 nm (Figure 2A). Under excitation at 580 nm, fluorescence spectra measurements showed a clear emission peak at 670-680 nm and a slight red shift that were absent in control preparations (Figure 2B). To further characterize the *in situ* assembly of Au-antimiRNA NCs, cell lysates were examined by TEM and AFM. On TEM images, gold nanocluster complexes with diameters of approximately 2-3 nm were clearly visible in lysates from cells co-incubated with antimiR-155 (Figure 2C). This finding is consistent with data from of our previous report on gold nanocluster-DNA complexes [29]. Meanwhile, AFM topography data revealed that the cumulative height of Au-antimiR-155 NCs was 3.5 nm (Figure 2D, left and right panels). Moreover, the two individual components of the NCs (i.e. Au and the miRNA inhibitor) could be distinguished on the chain structure visualized in the phase diagram (Figure 2D, middle panel). Accordingly, 3D AFM rendering showed that HepG2 cells cultured solely with gold precursor solution (HAuCl_4_) contained particles sizes of only 1.7 nm (Supplementary Figure 1). More importantly, no Au-antimiRNA NCs were observed when miRNA inhibitors and gold precursors were simultaneously added to L02 cells under identical experimental conditions. Then, we used laser confocal fluorescence imaging to further typify the presence of Au-antimiRNA NCs in culture media from HepG2 cells (Figure 2E). Confirming that HepG2 cells can spontaneously form fluorescent gold nanoclusters, clear differences in intracellular fluorescence were noted between cells cultured with gold precursor plus antimiR-155 and those treated with gold precursor alone. In contrast, no fluorescent signals were recorded under any experimental condition in L02 cells. These data highlight specific differences between tumor and non-tumor cells in their ability to assemble Au-antimiRNA NCs and confirm the feasibility of our nanotherapeutic approach for antimiRNA delivery.

### Au-antimiR-155 NCs inhibit proliferation and trigger apoptosis in HCC cells

**Figure 3:**
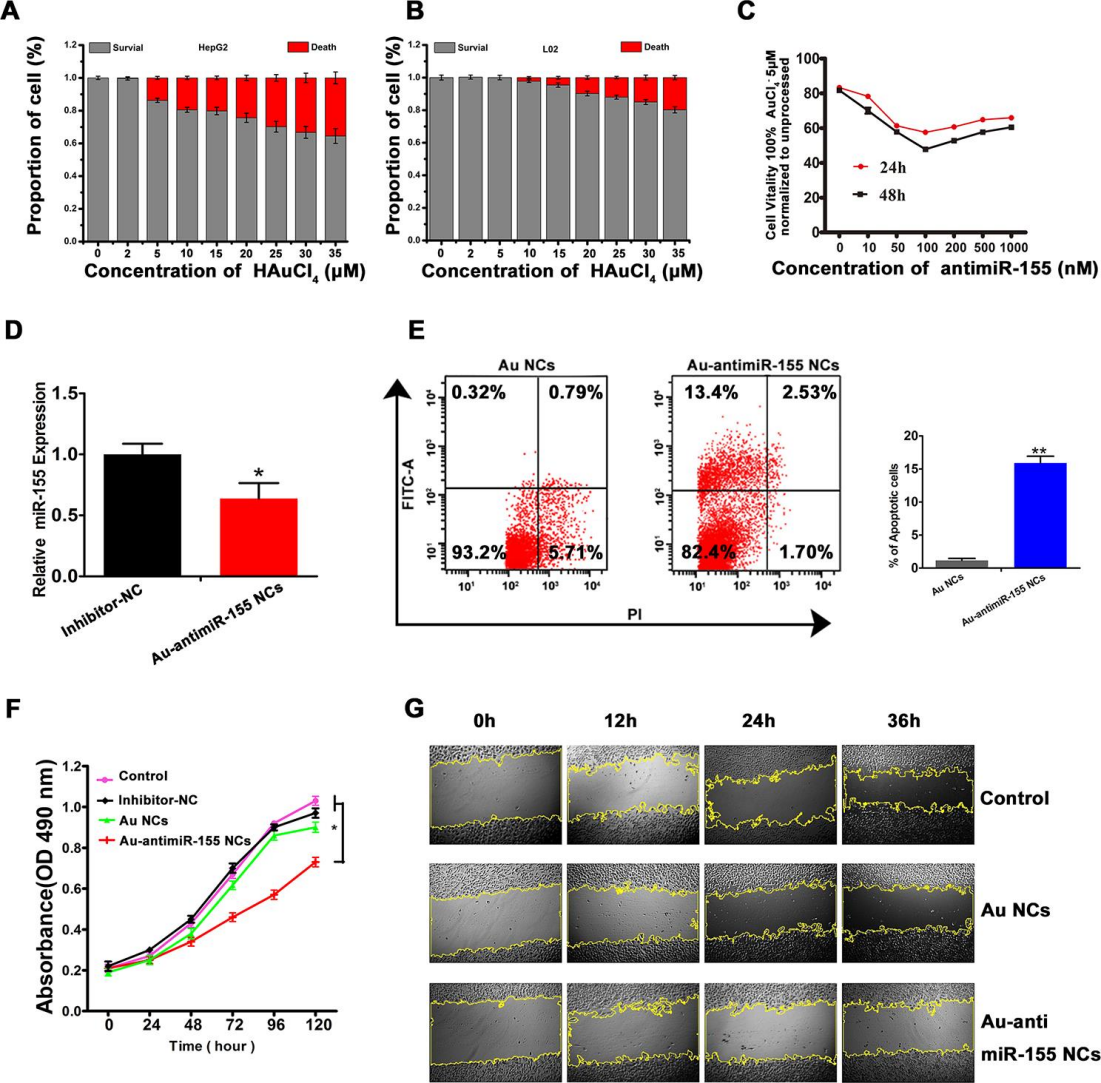
***In situ* self-assembled Au-antimir-155 NCs inhibit HepG2 cell growth.** (**A**, **B**) Evaluation of gold precursor cytotoxicity in HepG2 and L02 cells using the MTT assay. (**C**) Dose- and time-response analysis of the effect of different antimiR-155 concentrations on HepG2 viability (MTT assay). (**D**) Effect of Au-antimiR-155 on endogenous miR-155 expression in HepG2 cells. (**E**) Flow cytometry analysis of apoptosis in HepG2 cells that generated Au-antimiR-155 NCs or were treated with gold precursor alone (Au NCs). The left and right upper quadrants represent, respectively, early and late apoptotic cells (n=3). (**F**) Results of MTT proliferation assays in HepG2 cells under different conditions: Inhibitor-NC (inhibitor mimic negative control, 100 nM), *in situ* synthetized Au NCs (gold precursor, 5 μM), and *in situ* synthetized Au-antimiR-155 NCs (antimiR-155, 100 nM; gold precursor, 5 μM). (**G**) Images of wound-healing assays conducted in HepG2 cells under different experimental conditions (100×). Three biological replicates per experiment were assayed. **P<0.01, *P < 0.05.

We next explored the ability of miR-155 inhibitors to inhibit proliferation and trigger apoptosis in cultured HCC cells. Cytotoxicity MTT assays in HepG2 and L02 cells showed that HepG2 cell viability remained greater than 80% after incubation with gold precursor solutions ≤10 μM for 48 h (Figure 3A and 3B). Therefore, in subsequent experiments 5 μM HAuCl_4_ was used for *in situ* nanoconjugate assembly in HepG2 cells. Similarly, MTT assays were performed to optimize the concentration of miR-155 inhibitor used for the generation of Au-antimiRNA NCs. The results showed that the most obvious inhibitory effect was achieved at a concentration of 100 nM in cells co-incubated with 5 μM gold precursor (Figure 3C). After applying the latter conditions to generate self-assembled Au-antimiR-155 NCs in HepG2 cells, we demonstrated effective knockdown of miR-155 expression through qRT-PCR (Figure 3D), and induction of apoptosis using Annexin-V staining and flow cytometry (Figure 3E). Meanwhile, after 5-day incubation with either gold precursor, antimiR-155 negative control, or gold precursor plus antimiRNA-155, MTT assays showed that proliferation decreased significantly only in the latter group (Figure 3F). On the other hand, wound-healing assays demonstrated that the migratory capacity of HepG2 cells was significantly hampered by Au-antimiR-155 NCs formation (Figure 3G and Supplementary Figure 2).

### TLR3 is downregulated in HCC and is a binding partner of miR-155

**Figure 4:**
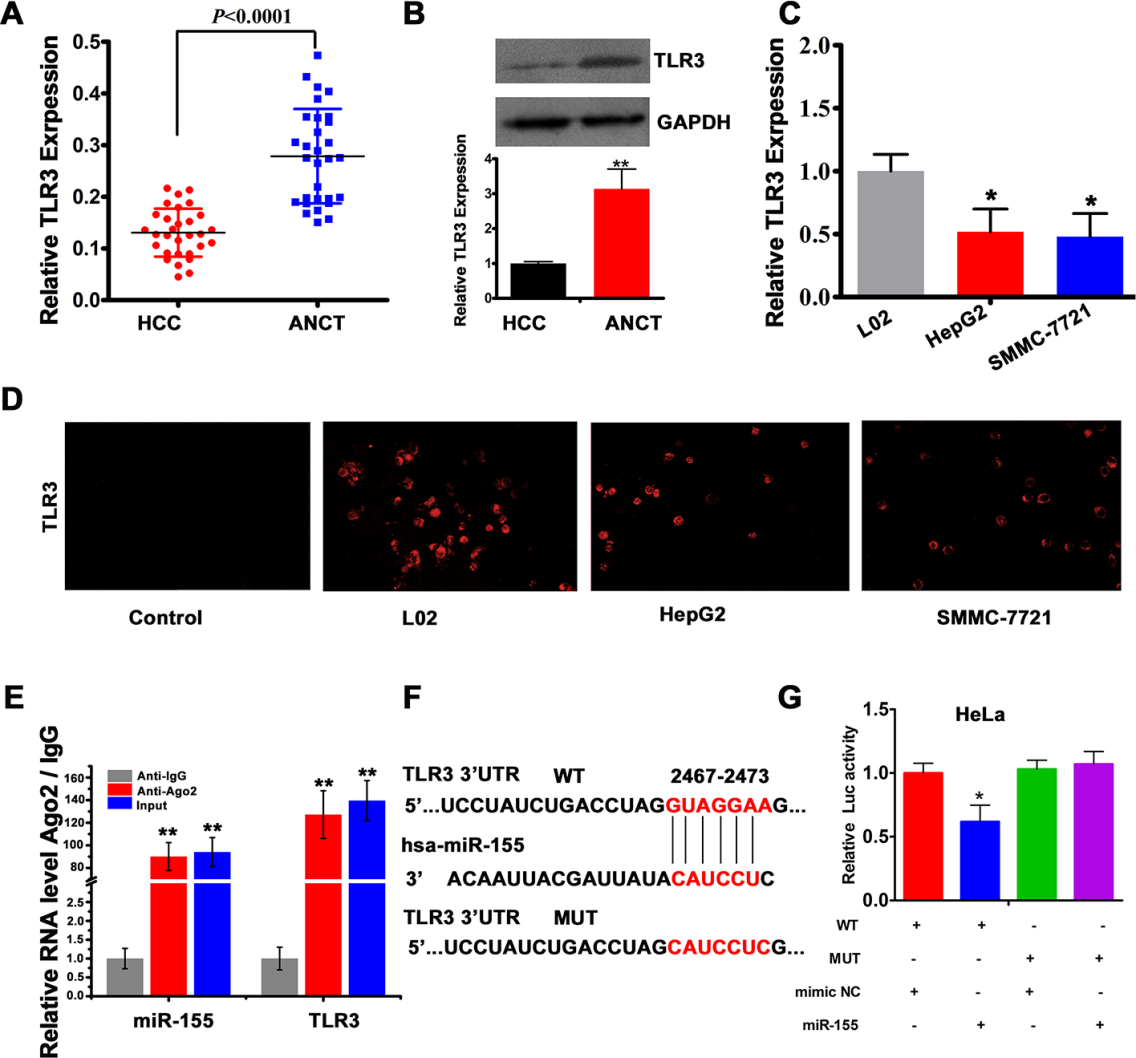
**TLR3 is a direct target of miR-155.** (**A**) qRT-PCR analysis of TLR3 expression in HCC tissues and ANCTs (n=30). (**B**) Western blot and densitometric quantification analysis of TLR3 expression in HCC tissues and ANCTs. (**C**) qRT-PCR analysis of TLR3 expression in HCC and normal liver cells (n=3). (**D**) TLR3 immunofluorescence in L02, HepG2 and SMMC-7721 cells. (**E**) RIP assay results showing co-precipitation of miR-155 and TLR3 by an Ago2 antibody in HepG2 cells. Verification of RIP products by qRT-PCR is also shown. (**F**) Schematic representation of the miR-155 binding site in the 3'-UTR region of TLR3 (WT, wild type; MUT, mutated type). (**G**) Luciferase activity assay results demonstrating direct targeting of the 3'UTR of TLR3 by miR-155 (n=3). **P<0.01, *P < 0.05.

To explore the mechanism by which miR-155 influences HCC progression, we assessed the expression of one of its target genes, i.e. TLR3, in clinical HCC specimens. Results of qRT-PCR and western blot analyses showed that TLR3 mRNA and protein levels were reduced in HCC tissues compared to ANCTs (Figure 4A and 4B). Similarly, TLR3 mRNA levels were clearly lower in HepG2 and SMMC-7721 cells compared to L02 cells (Figure 4C). In addition, immunofluorescence experiments confirmed reduced TLR3 expression in HCC cells, and evidenced cytoplasmic compartmentation for the TLR3 protein (Figure 4D). To confirm the predicted interaction between miR-155 and TLR3, we performed RNA immunoprecipitation (RIP) and dual-luciferase reporter assays. RIP results revealed that miR-155 and TLR3 were co-immunoprecipitated by an Ago2 antibody in HepG2 cells, indicating that both transcripts colocalized to the RNA-induced silencing complex (RISC). Subsequently, we confirmed through qRT-PCR that the precipitated products were indeed miR-155 and TLR3 (Figure 4E). After co-transfection of HeLa cells with luciferase constructs containing either the wild-type (WT) or a mutated form (MUT) of the 3’-UTR of the TLR3 mRNA (Figure 4F) and miR-155 mimics, dual-luciferase reporter assays showed decreased luciferase activity for the WT vector. Confirming the specificity of this interaction, no signal reduction was observed in cells co-transfected with control miRNA (mimic NC) or in those co-expressing the MUT construct (Figure 4G).

### MiR-155 is a negative regulator of TLR3 expression

**Figure 5:**
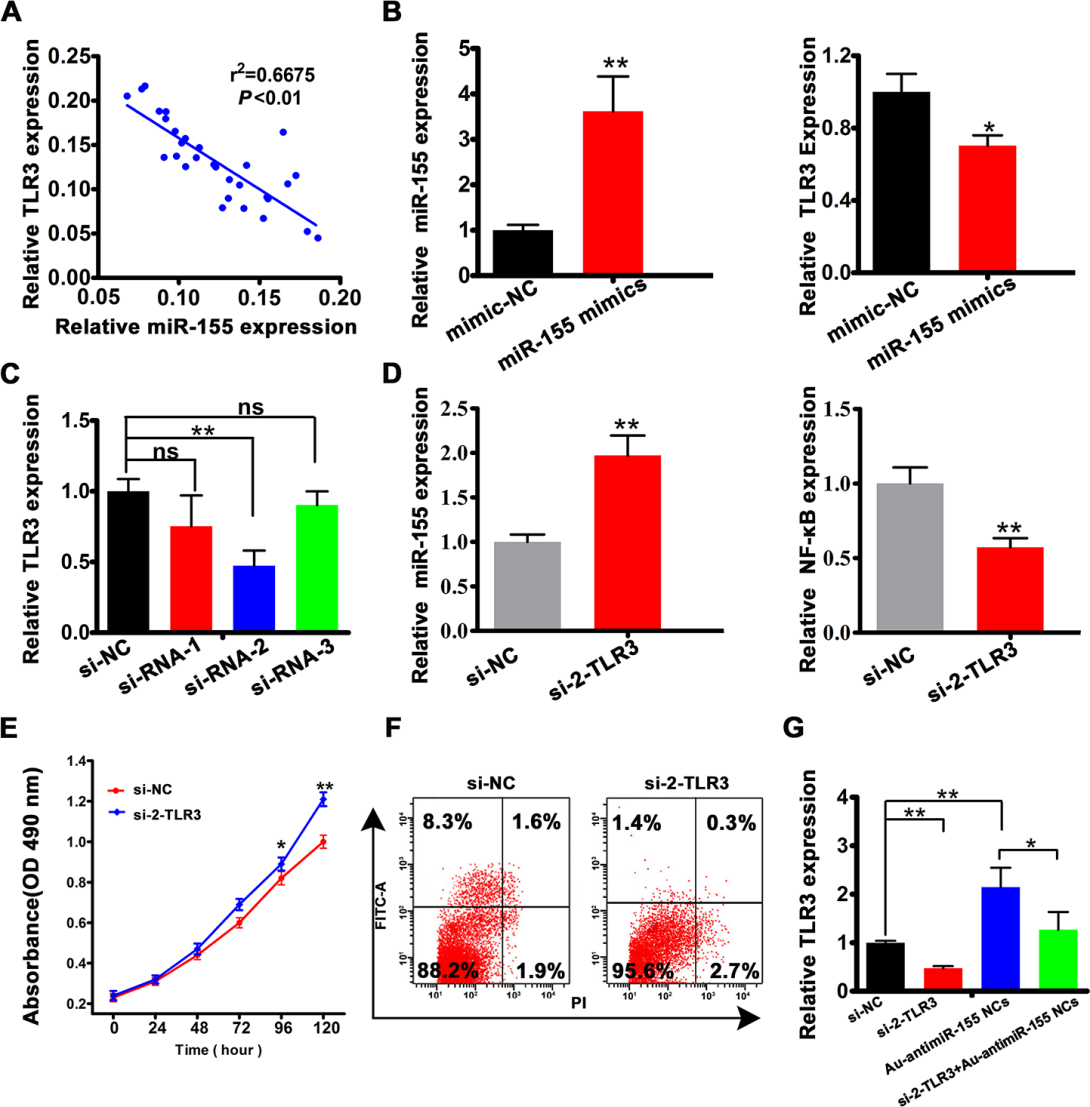
**miR-155 is a negative regulator of TLR3 expression.** (**A**) Spearman's correlation analysis of the relationship between TLR3 and miR-155 levels in HCC tissues. (**B**) Analysis of miR-155 and TLR3 expression by qRT-PCR in HepG2 cell transfected with miR-155 mimics. (**C**) qRT-PCR analysis of TLR3 expression in HepG2 cells transfected with control siRNA (si-NC) or with 3 siRNA variants targeting TLR3. (**D**) Detection of miR-155 and NF-κB by qRT-PCR in HepG2 cells transfected with si-2-TLR3. (**E**, **F**) Results of cell viability (MTT) and apoptosis (Annexin V-FITC) assays conducted in HepG2 cells transfected with si-2-TLR3 or si-NC. (**G**) Quantification of TLR3 mRNA levels in HepG2 cells following si-2-TLR3-mediated TLR3 knockdown with or without concurrent miR-155 inhibition. Data are presented as the mean ± SD; n = 3 biologically independent samples. *P<0.05, **P<0.01; ns, no significant difference (Student’s t-test).

To further assess the clinical significance of the miR-155/TLR3 interaction, we first conducted Spearman’s correlation analysis in clinical HCC samples. As expected, a negative correlation between miR-155 and TLR3 expression was observed (r^2^ = 0.6675, Figure 5A). To further characterize this relationship, we transfected HepG2 cells with miR-155 mimics. As a result, TLR3 expression was significantly decreased (Figure 5B). To explore the functional role of TLR3, the knockdown efficiency of three TLR3-targeted siRNAs was tested. The results indicated that siRNA-2 was the most effective one (Figure 5C), and was therefore selected for use in subsequent experiments. To evaluate the ability of TLR3-siRNA-2 (si-2-TLR3) to block TLR3 activation, we assessed the expression of NF-κB, a major transcriptional downstream effector of TLR pathways [30, 31]. As shown in Figure 5D, TLR3-siRNA-2 transfection significantly increased the expression of miR-155 and decreased the expression of the NF-κB subunit p65. Meanwhile, additional in vitro assays revealed that TLR3 knockdown increased proliferation (Figure 5E) and inhibited apoptosis (Figure 5F and Supplementary Figure 3) in HepG2 cells. Importantly, TLR3 expression was significantly increased in cells producing Au-antimiR-155 NCs, while further expression assays showed that concurrent inhibition of miR-155 counteracted the inhibitory effect of si-2-TLR3 on TLR3 expression (Figure 5G). These results strongly suggest that miR-155 inhibits TLR3 by direct binding and indicate that Au-antimiR-155 NC formation can reactivate TLR3 expression in HepG2 cells.

### Au-antimiR-155 NCs activate pro-apoptotic TLR3 signaling

**Figure 6:**
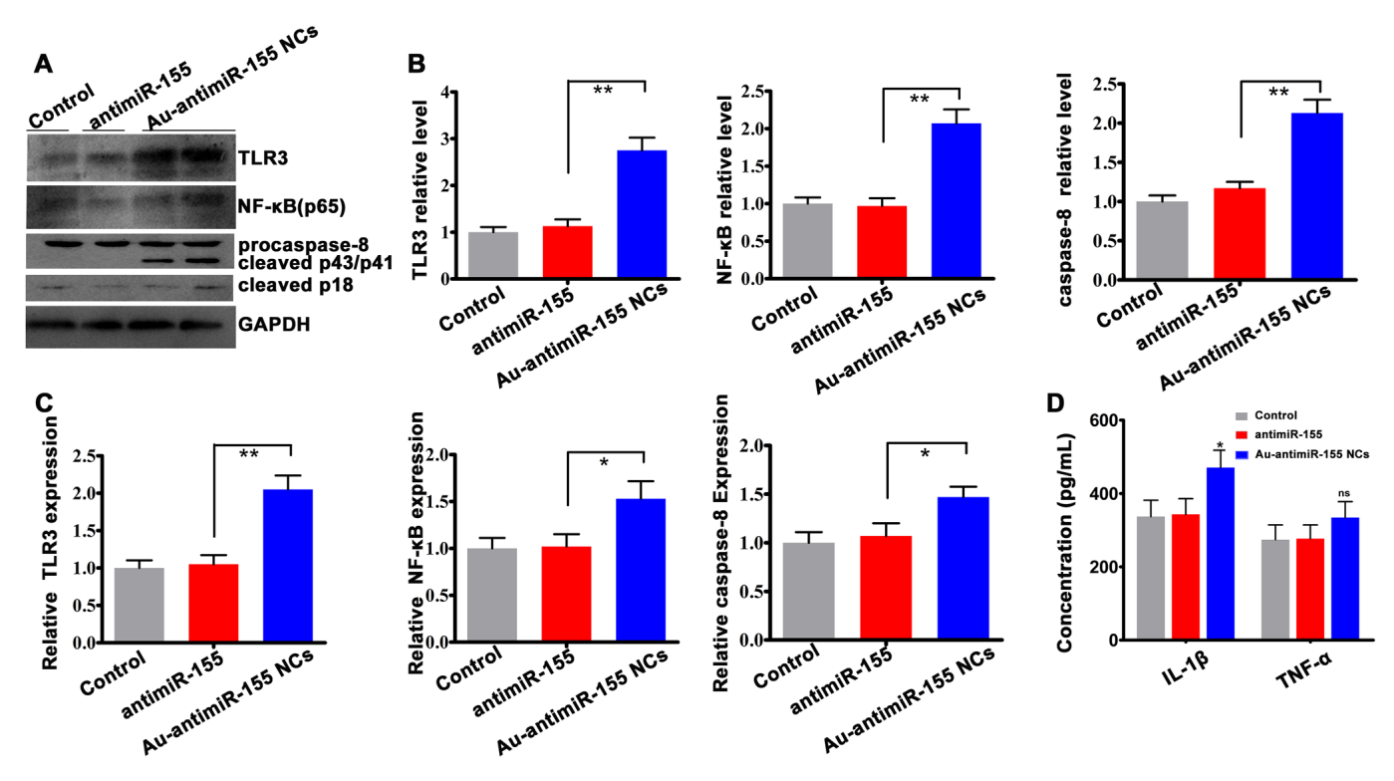
**Activation of TLR3 signaling by Au-antimiR-155 NCs.** (**A**) Western blotting analysis of TLR3, NF-αB (p65), and both full-length and cleaved caspase-8 expression in HepG2 cells treated with antimiR-155, gold precursor plus antimiR-155, or the respective controls (n=3). (**B**) Densitometric quantification of data shown in (**A**). (**C**) qRT-RCR analysis of TLR3, p65, and caspase-8 expression in HepG2 cells (n=3). (**D**) Quantification of tumor necrosis factor-α (TNF-α) and interleukin-1β (IL-1β) secretion by ELISA. HepG2 cells were co-incubated with gold precursor and antimiR-155 for 24 h. **P< 0.01; *P < 0.05; ns, no significant difference.

HepG2 cells that produced Au-antimiRNA NCs over 24 h showed significantly increased expression of TLR3 and p65 at the protein (Figure 6A and 6B) and mRNA (Figure 6C) levels. Past research showed that TLR3 signaling in HepG2 cells leads to apoptosis [20]. Since caspase-8 is an important mediator of death receptor-mediated apoptosis, we assessed caspase-8 expression in HepG2 cells 24 h after induction of Au-antimiR-155 NCs synthesis. Results showed that both full-length pro-caspase-8 and active (cleaved) caspase-8 levels were increased at the protein (Figure 6A and 6B) and mRNA (Figure 6C) levels. Caspase-8 mediates processing of pro-IL-1β into its mature 17 kDa form [32]. Using Enzyme linked immunosorbent assay (ELISA), we found that the assembly of Au-antimiR-155 NCs promoted both IL-1β and TNF-α expression, although a significant increase was noted only for the former cytokine (Figure 6D). These findings suggest that the anti-proliferative and pro-apoptotic effects of Au-antimiR-155 NCs in HCC cells are mediated by activation of TLR3 signaling, leading to upregulation of NF-κB expression, activation of caspase-8, and induction of IL-1β.

### Clinical correlation of miR-155 and TLR3 expression in HCC patients

Lastly, we analyzed the potential association between miR-155 and TLR3 expression patterns and HCC progression and prognosis (Table 1). To this end, we categorized HCC patients into two groups (i.e. high and low expression) based on miR-155 and TLR3 mRNA expression levels relative to control ANCT expression data. Correlation analyses showed that high miR-155 expression was associated with tumor size (*P* = 0.007), venous invasion (*P* = 0.024), metastasis (*P* = 0.039), and BCLC stage (*P* = 0.024). In turn, low TLR3 expression was significantly correlated with tumor size (*P* = 0.007) and venous invasion (*P* = 0.016). Next, univariate and multivariate Cox regression analyses were performed to assess the prognostic value of miR-155 for overall survival (Table 2). Both analyses showed that miR-155, venous invasion, and distant metastasis were significant predictors of poor OS in HCC patients (*P*<0.05).

**Table 1:** Correlations between miR-155 expression and clinicopathological characteristics in 30 HCC tissues.

**Characteristic**	**n**	**miR-155 expression**		***P* value**	**TLR3 expression**		***P* value**
		**Low**	**High**		**Low**	**High**	
All case	30	14	16		18	12	
Gender				0.378			0.660
Male	24	10(41.7%)	14(58.3%)		15(62.5%)	9(37.5%)	
Female	6	4(66.7%)	2(33.3%)		3 (50.0%)	3(50.0%)	
Age				0.675			0.669
≥60	23	10(43.5%)	13(56.5%)		13(56.5%)	10(43.5%)	
<60	7	4(57.1%)	3(42.9%)		5(71.4%)	2(28.6%)	
liver disease				0.157			0.622
HBV	25	10(40.0%)	15(60.0%)		14(56.0%)	11(44.0%)	
HBV+ others	5	4 (80.0%)	1 (20.0%)		4(80.0%)	1 (20.0%)	
Liver cirrhosis				0.236			1.000
With	21	8(38.1%)	13(61.9%)		13(61.9%)	8(38.1%)	
Without	9	6(66.7%)	3(33.3%)		5(55.6%)	4(44.4%)	
Family history of liver disease				0.491			0.176
with	13	7(53.8%)	6(46.2%)		6(46.2%)	7(53.8%)	
without	17	7(41.2%)	10(58.8%)		12(70.6%)	5(29.4%)	
Tumor number				1.000			0.392
Single	23	11(47.8%)	12(52.2%)		15(65.2%)	8(34.8%)	
Multiple	7	3(42.9%)	4(57.1%)		3(42.9%)	4(57.1%)	
Tumor size (cm)				0.007**			0.007*
>5	12	2(16.7%)	10(83.3%)		11(91.7%)	1(8.3%)	
≤5	18	12(66.7%)	6(33.3%)		7(38.9%)	11(61.6%)	
TNM stage				0.232			0.709
I + II	18	10(55.6%)	8(44.4%)		10(55.6%)	8(44.4%)	
III + IV	12	4(33.3%)	8(66.7%)		8(66.7%)	4(33.3%)	
Venous invasion				0.024*			0.016*
with	13	3(23.1%)	10(76.9%)		11(84.6%)	2(15.4%)	
without	17	11(64.7%)	6(35.3%)		7(41.2%)	10(58.8%)	
Distant metastasis				0.039*			0.099
with	8	1(12.5%)	7(87.5%)		7(87.5%)	1(12.5%)	
without	22	13(59.1%)	9(40.9%)		11(50.0%)	11(50.0%)	
BCLC stage				0.024*			0.722
A	17	11(64.7%)	6(35.3%)		9(52.9%)	8(47.1%)	
B+C+D	13	3(23.1%)	10(76.9%)		9(69.2%)	4(30.8%)	
AFP levels (U/L)				0.602			0.632
>20	26	13(50.0%)	13(50.0%)		15(57.7%)	11(42.3%)	
≤20	4	1(25.0%)	3(75.0%)		3(75.0%)	1(25.0%)	

*P<0.05, **P<0.01; HBV: hepatitis B virus; TNM: tumour, node and metastasis; BCLC: Barcelona Clinic Liver

Cancer; AFP: alpha-fetoprotein.

**Table 2:** Univariate and multivariate Cox regression analyses of overall survival in HCC patients.

**Characteristics**	**B**	**SE**	**Wald**	***P* value**	**OR**	**95.0% CI**	
						**Lower**	**Upper**
Univariate Analysis (Cox: Enter)							
Gender (Female / Male)	-0.431	0.706	0.372	0.542	0.650	0.163	2.595
Liver cirrhosis (Absence / Presence)	-0.227	0.523	0.189	0.664	0.797	0.286	2.219
Family history of liver disease (with/without)	0.044	0.463	0.009	0.924	1.045	0.422	2.588
Tumor number (Single / Multiple)	-0.724	0.499	2.106	0.147	0.485	0.182	1.289
Tumor size (≤5cm / >5cm)	0.033	0.459	0.005	0.942	1.034	0.420	2.543
TNM stage (I, II / III, IV)	0.560	0.488	1.314	0.252	1.750	0.672	4.559
Venous invasion (with/without)	1.401	0.586	5.717	0.017*	4.059	1.287	12.798
Distant metastasis (with/without)	1.264	0.388	10.625	0.001*	3.541	1.656	7.574
BCLC stage (A/B,C,D)	-0.089	0.484	0.034	0.854	0.915	0.354	2.364
miR-150 (Low / High)	1.808	0.740	5.978	0.014*	6.100	1.432	25.996
Multivariate Analysis (Cox: Forward LR)							
Venous invasion (with/without)	1.134	0.516	4.824	0.028	3.109	1.130	8.554
Distant metastasis (with/without)	1.119	0.356	9.874	0.002	3.063	1.524	6.156
miR-150 (Low / High)	1.890	0.533	12.589	0.000	6.619	2.330	18.800

*P<0.05; B: beta-coefficients; SE: standard error; Wald: Wald test; OR: odds ratio;

CI: confidence interval.

## DISCUSSION

Although several studies advanced the potential of TLR3 as a therapeutic target for hepatoma, melanoma, and clear cell renal carcinoma [20, 33–35], the mechanisms mediating its antitumor effects remain insufficiently characterized [36]. On the other hand, the discovery of numerous miRNAs functioning as critical regulators of cancer-related processes has stimulated intense research to define the therapeutic potential of miRNA modulation to modify the behavior of cancer cells [37]. Previous research suggested that upregulation of miR-155 contributes to HCC progression, while low TLR3 expression is associated with poor prognosis in HCC patients and may mediate apoptosis escape in HCC cells [13, 17, 38]. In this work, we evaluated the ability of *in situ* self-assembled Au-antimiR-155 NCs to inhibit miR-155 activity, and the concomitant impact on the expression and function of TLR3, a predicted miR-155 target, in cultured HCC cells.

Using UV-Vis spectrometry, fluorescence spectra measurements, and TEM and AFM imaging, we described a method to generate *in situ* self-assembled Au-antimiRNA NCs and investigated the potential of this nanotherapeutic approach to halt HCC cell replication. Importantly, self-assembly of Au-antimiR-155 NCs was not observed in normal L02 hepatocytes, which suggests that this process is tumor cell-specific and may thus be effective for *in vivo* delivery of miRNA-targeted cancer therapy [25]. Following alternative or simultaneous incubation of HepG2 cells with miR-155 inhibitor and gold precursor solution (HAuCl_4_), successful self-assembly of fluorescent Au-miRNA NCs was determined based on analysis of the various conformational states of the resulting biosynthetic products by TEM and AFM. On the other hand, we confirmed that miR-155 was upregulated in a subset of clinical HCC specimens and found that its expression is negatively correlated with that of TLR3. We also proved through RIP the interaction of miR-155 and TLR3 at the RISC and confirmed, using luciferase reporter assays, that miR-155 directly binds TLR3 and downregulates its expression in cultured HepG2 cells. Importantly, we verified the ability of self-assembled Au-antimiR-155 NCs to inhibit proliferation and migration and to promote apoptosis in HepG2 cells.

We showed that knockdown of miR-155 expression by Au-antimiR-155 NCs increased the expression of TLR3, and effect of TLR3 silencing was counteracted by concurrent inhibition of miR-155. Interestingly, siRNA-mediated silencing of TLR3 led to a significant increase in miR-155 mRNA expression. However, since previous research suggested that TLR signaling induces miR-155 expression [39–40], this finding requires further experimental analysis. Contrary to the effects of miR-155 silencing, TLR3 knockdown increased the proliferative ability and inhibited apoptosis in HepG2 cells, an effect likely related to the concomitant inhibition of NF-κB transcription revealed by qRT-PCR. TLR3 signaling was shown to trigger MyD88-independent NF-κB production [31] and to mediate, alternatively, inflammatory and apoptotic responses [30]. Previous studies indicated that TLR3 signaling preferentially activates the apoptotic pathway in HCC [20] and other tumor cells [34, 41]. Moreover, NF-κB was shown to induce pro-IL-1β expression in hepatocytes, leading to cell death upon caspase-8-mediated activation [30, 32, 42]. Induction of inflammatory and apoptotic pathways was shown also to occur following TLR3 stimulation via activation of NF-κB and upregulation of caspase-8 and IFN-γ in HepG2.2.15 cells [43]. Meanwhile, Vince et al. reported that inhibition of Inhibitor of Apoptosis Proteins (IAPs) results in the processing and secretion of IL-1β through RIP3-mediated caspase-1-and caspase-8-dependent pathways [44]. We found that in addition to NF-κB, both full-length pro-caspase-8 and cleaved, active caspase-8 was upregulated in HepG2 cells producing Au-antimiR-155 NCs, and these changes were accompanied by increased release of bioactive IL-1β and apoptosis induction. These results suggest that *in situ* self-assembly of Au-antimiR-155 NCs can be an effective strategy to stimulate the expression of TLR3 and restrict HCC cell proliferation through apoptosis induction.

Consistent with previous data, Kaplan-Meier analysis of HCC patients revealed shorter OS in those with high, rather than low, miR-155 expression [13]. In turn, ROC/AUC analysis demonstrated acceptable diagnostic performance (AUC = 0.778) for high miR-155 expression in HCC, suggesting that miR-155 expression could serve as an independent prognosis predictor for HCC patients. Multiple clinical and pathological factors are evaluated to determine HCC severity, to predict prognosis, and to determine the best treatment options. We found that high miR-155 expression was positively associated with tumor size, venous invasion, metastasis, and BCLC stage, but was not correlated with TNM stage. Meanwhile, low TLR3 expression was inversely correlated with tumor size and venous infiltration. Together with the above experimental results, these clinical findings provide further, reliable bases for the use of miR-155-targeted therapies to upregulate TLR3 and restrict, via TLR3-induced apoptosis, HCC progression.

**Figure 7:**
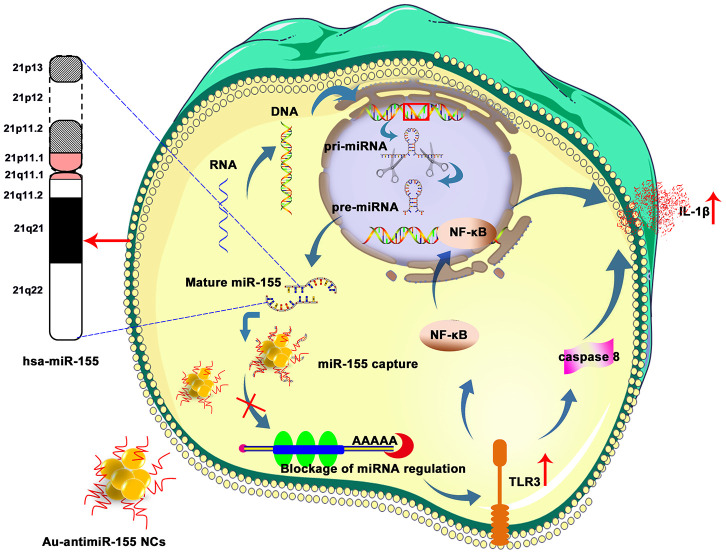
**Schematic representation of Au-antimiR-155 NC-mediated activation of growth inhibitory/pro-apoptotic TLR3 signaling in HepG2 cells.** De-repression of miR-155-induced TL3R silencing by Au-antimiR-155 NCs promotes apoptosis via TLR3 activation, NF-κβ transcription, caspase-8 activation, and IL-1β release.

In conclusion, we describe an *in situ* self-assembled Au-antimiRNA NC delivery system that efficiently downregulates miR-155 and activates TLR3 expression and signaling in HCC cells. This action leads to upregulation of NF-κB, caspase-8, and IL-1β expression. and cell death via apoptosis (Figure 7). Furthermore, data from our Kazakh patient cohort verified that miR-155 and TLR3 might serve as a novel biomarker to predict the progression and prognosis of HCC. While our *in vitro* findings indicate that miR-155 can be efficiently targeted by self-assembled Au-antimiRNA NCs in HCC cells, further experiments assessing specific tumor delivery and anti-tumor effects *in vivo* are needed to confirm the potential applicability of our nanotherapeutic approach.

## MATERIALS AND METHODS

### Patients and specimens

HCC tissues and adjacent non-cancer tissues (ANCTs) (n=30) were obtained from patients diagnosed with HCC in the department of Hepatobiliary Surgery, Xinjiang Yili Kazakh Autonomous Prefecture Friendship Hospital, from 2014 to 2018. The patients were 54-87 years old (mean age 67 years). The study was approved by the Ethics Committee of Xinjiang Yili Friendship Hospital, and each subject signed an informed consent form. All tissue specimens were frozen in liquid nitrogen immediately after resection and kept at -80°C until analysis. For each patient, demographic and epidemiological data (age, gender, genetic history, tobacco and alcohol use, etc.) and biochemical indicators were collected (Table 1 and Supplementary Table 1).

### Quantitative real-time PCR analysis

Total tissue RNA was extracted using Trizol reagent (Invitrogen, Carlsbad, CA, USA) and sample concentration and purity verified through standard methods. Analysis of miR-155 expression was performed using a Hairpin-it qRT-PCR kit (GenePharma, Shanghai, China) according to the manufacturer’s instructions using U6 snRNA as internal control. TLR3 expression was assayed with a SYBR Green PCR kit (GenePharma) with GAPDH as internal control. Reactions containing neither reverse transcriptase nor template were used as negative controls. PCR reactions were carried out on an Applied Biosystems 7500 Real-Time PCR system. Each assay was repeated 3 times. Relative gene expression was quantified by the 2^-ΔΔCT^ method. Convergent primers were used to detect mRNAs and stem-loop RT primers were used to detect miR-155. Primer sequences are shown in Supplementary Table 2.

### Cell culture

HepG2, SMMC-7721, and HeLa cells were purchased from the ATCC (Manassas, VA, USA). The L02 cell line (human fetal hepatocytes) was obtained from the Third Military Medical University (Chongqing, China). All cells were maintained in high glucose-DMEM (HyClone, China), supplemented with 10% fetal bovine serum and 1% penicillin/streptomycin (HyClone, Australia) at 37 °C in a 95% humidified atmosphere with 5% CO_2_.

### *In situ* self-assembly and extraction of Au-antimiRNA NCs

HepG2 cells were plated and allowed to adhere for 6-12 h. Then, gold precursor solution (HAuCl_4_; Sigma Aldrich, USA; pH=7.2; CAS: 16903-35-8) was added to the culture to a final concentration of 5 μM. Next, a miRNA inhibitor (antimiR-155, single-stranded RNA, 100 nM; GenePharma) was added to the medium. After incubation for 24 or 48 h, the medium was discarded and the cells were harvested with trypsin, washed in PBS, centrifuged at 1500 rpm for 3 min, and resuspended in deionized water. Following cell lysis through repeated freeze-thaw cycles, lysates were centrifuged at 2500 rpm for 20 min and the supernatants filtered (0.22 μm) to obtain cell extracts for downstream analyses of Au-antimiR-155 NC formation.

### Characterization of *in situ* self-assembled Au-antimiRNA NCs

A UV-Vis-NIR spectrophotometer (Thermo Scientific Evolution 260, USA) and a fluorescence spectrometer (RF-5301PC, Japan) were used, respectively, for UV-Vis absorption and fluorescence spectra determinations. A JEM-2100 transmission electron microscope (TEM) was utilized to characterize size distribution of the *in situ* biosynthesized Au-antimiRNA NCs. An atomic force microscope (AFM) (Bruker Dimension Icon, USA) was used to study the morphology of Au-antimiRNA NCs. For nanocluster imaging of cell culture media, laser confocal dishes and a confocal microscope with a 488 nm laser (Andor Revolution XD system) were used for imaging 12 or 24 h after co-incubation of gold precursors and miR-155 inhibitors. The presence of biosynthesized Au-antimiRNA NCs was analyzed using a 20×IR coated objective (Nikon Eclipse C1, Japan). Each experiment was conducted three times.

### Cell proliferation assay

Cell proliferation was measured using the 3-(4,5-dimethylthiazol-2-yl)-2, 5-diphenyltetrazolium bromide (MTT) assay. HepG2 cells in logarithmic growth phase were seeded in 96-well plates at a density of 4×103 cells/well in 200 μL of complete medium. After 24 h, the cells were rinsed with DMEM and incubated with antimiR-155 (100 nM) and gold precursor (5 μM) for 0, 1, 2, 3, 4, or 5 days. The MTT assay was then performed and cell growth curves were plotted for each time point, using three biological replicates per experiment.

### Wound healing assay

Wound healing assays were performed on HepG2 cells plated on 6-well culture plates (5×105/well). Cells were co-incubated with gold precursor (2 μM) and antimiR-155 (25 nM). When cell confluence reached 80%-90%, a thin wound was scratched through the midline of the culture with a pipette tip and the medium replaced with low-serum DMEM. Images were captured over 36 h at fixed intervals and analyzed by Image J software.

### Apoptosis assay

To assess the effect of Au-antimiR-155 NCs on apoptosis, HepG2 cells were plated in 6-well plates (1 × 105 cells/well) and incubated with gold precursor (5 μM) and antimiR-155 (100 nM) for 6-12 h. To assess the impact of TLR3 silencing on apoptosis, some cultures were co-transfected for 12 h with si-2-TLR3 (100 pmol) or scrambled negative control (si-NC, 100 pmol) siRNAs using 5 μl Lipofectamine 2000 (Invitrogen, CA, USA). The cells were then detached, mixed thoroughly with 5 μL of Annexin V-FITC in 500 μL of binding buffer, and incubated for 15 min at room temperature in the dark. Following addition of 5 μL of PI, apoptotic rates were determined using a flow cytometer and FlowJo software.

### Luciferase reporter assay

Binding sites for miR-155 in the TLR3 mRNA sequence were predicted using TargetScan Human 7.1 database. Constructs containing the wild-type (WT) or a mutant (MUT) 3′ UTR sequence of TLR3 mRNA were inserted into the pmiR-GLO dual-luciferase vector (GenePharma). HeLa cells were co-transfected with miR-155 mimics or mimic NC along with WT or MUT vectors using Lipofectamine 2000. Cells were collected 48 h after transfection and luciferase activity was detected using a dual-luciferase reporter assay system (Promega, Madison, WI, USA) according to the manufacturer’s instructions. Renilla activity was calculated for normalization. Each experiment was performed in triplicate.

### RNA immunoprecipitation (RIP)

RIP experiments were performed using a Magna RIP™ RNA-Binding Protein Immunoprecipitation Kit (Millipore, USA) according to the manufacturer’s instructions. In brief, 2 × 10^7^ HepG2 cells were trypsinized, rinsed twice with ice-cold PBS, and resuspended in an equal volume of RIP lysis buffer. Next, 5 μg of Ago2 antibody was added to the magnetic beads, and the mixture was incubated at room temperature in RIP wash buffer with continuous stirring for 30 min. The immunoprecipitates were digested in proteinase K buffer, and RNA was extracted for qRT-PCR analysis. All reagents and consumables were DNase- and RNase-free, and each experiment was repeated three times.

### Immunofluorescence staining

Cells grown on coverslips were fixed with acetone/methanol (1:1) for 20 min on ice and washed in 3 changes of PBS (5 min each). Blocking was performed at room temperature for 30 min, in 3 changes of 1% BSA/0.1% Tween 20/PBS. An anti-TLR3 antibody (ab62566, Abcam; Cambridge, UK), diluted 1:200 in blocking buffer, was applied overnight at 4°C. After 3 washes in blocking buffer, a donkey anti-rabbit IgG-labeled secondary antibody was added in blocking buffer for 1 h at room temperature. Cells were washed in blocking buffer, mounted in antifade medium, and visualized and photographed using fluorescence microscopy.

### siRNA design and synthesis

The design and synthesis of three siRNAs directed against TLR3 mRNA (Supplementary Table 3) was performed by RiboBio Co., Ltd (Guangzhou, China). Lipofectamine 2000 was used for siRNA transfection, according to the manufacturer’s protocol.

### Western blotting

Total cell lysates were prepared and subjected to SDS-PAGE according to standard procedures. Briefly, protein samples were mixed with protein loading buffer and heated at 100°C for 3-5 min to completely denature the proteins. SDS-PAGE was carried out using 10%/5% acrylamide separating/stacking gels followed by electrotransfer onto PVDF membranes. The membranes were blocked with 5% nonfat dry milk-blocking solution for 1 h at room temperature, add rabbit antibodies against TLR3, NF-κB (p65), and caspase-8 (Biosen Biotechnology Co., Beijing, China) were applied at 4°C overnight (See Supplementary Table 4 for details). Goat anti-rabbit IgG labeled with horseradish peroxidase (HRP) was then applied for 2 h, and signals detected on a Bio-Rad chemiluminescence imager after addition of chemiluminescent substrate. Bands’ optical density was determined using Quantity One software and normalized to GAPDH (internal control). Three biological replicates per sample were assayed.

### Kaplan–Meier analysis

Overall survival of HCC patients was evaluated using Kaplan-Meier analysis. Cox proportional hazard regression was applied to assess progression and prognosis. The diagnostic predictive value of miR-155 expression was tested via ROC analysis. Based on miR-155/TLR3 expression levels relative to ANCT, HCC patients were divided into two groups (High/Low expression). The log-rank test was used to evaluate differences between curves. Significance was defined at the 0.05 level.

### Enzyme linked immunosorbent assay (ELISA)

Quantitative measurements of TNF-α (E-EL-H0109c, Elabscience, China) and IL-1β (E-EL-H0149c, Elabscience) released into cell culture media were performed by ELISA according to manufacturer’s directions. The detection range was 7.81- 500 pg/mL with a sensitivity of 4.69 pg/mL.

### Statistical analyses

The data were tested for normality and homogeneity of variance and displayed as the mean ± SD of at least three independent experiments. Comparisons between groups were made with Student's *t*-test or one-way ANOVA. The Kaplan-Meier method was used to generate overall survival curves, and the log-rank test used for comparisons. Correlation analysis between two sets of data was performed by Spearman’s correlation analysis. Statistical analyses were performed with GraphPad Prism software (Version 5.01; GraphPad, San Diego, CA, USA) and SPSS for Windows version 17.0 (SPSS Inc, Chicago, IL, USA). *P*<0.05 indicated a statistically significant difference.

### Ethics approval

The study was approved by the Ethics Committee of Xinjiang Yili Friendship Hospital, and Shunde Hospital of Southern Medical University. Informed consent was obtained from all participants included in the study.

## Supplementary Material

Supplementary Figures

Supplementary Tables
