# Supplementary material for: In situ self-assembly of Au-antimiR-155 nanocomplexes mediates TLR3-dependent apoptosis in hepatocellular carcinoma cells
Source: Aging (Albany NY). 2020 Nov 5;13(1):241–61. doi: 10.18632/aging.103799 (PMC7834998; doi:10.18632/aging.103799)
Supplement: Supplementary Figures [file aging-13-103799-s001.pdf]

SUPPLEMENTARY FIGURES

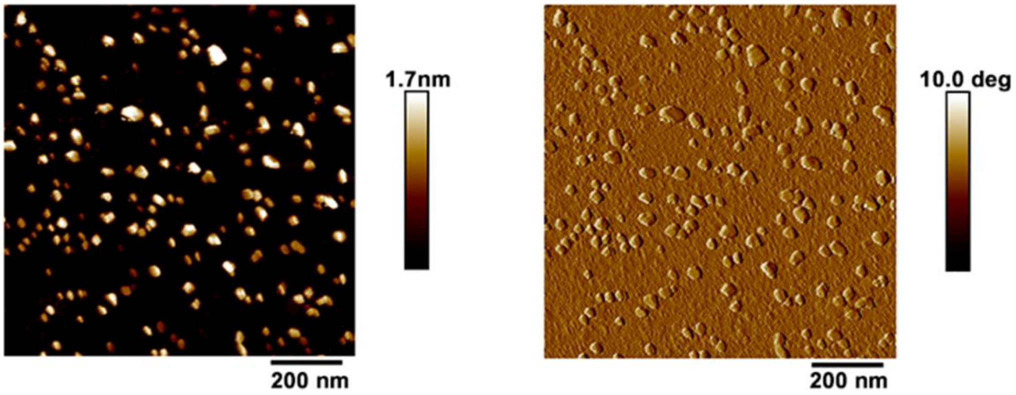

Supplementary Figure 1. Typical AFM images (left panel) and phase graph (right panel) of the biosynthesized Au NCs extracted from HepG2 cells after cultured with gold precursor alone.

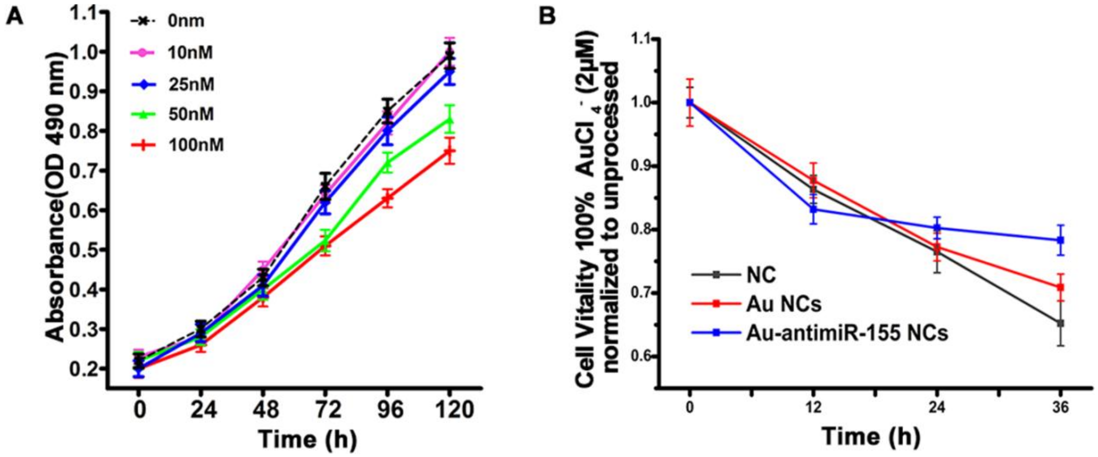

Supplementary Figure 2. (**A**) 5d-MTT was used to select the concentration of antimiR-155 to use for scratch-healing experiment. (**B**) Analysis of a scratch-healing experiment with HepG2 cells under different conditions. Three biological replicates per experiment.

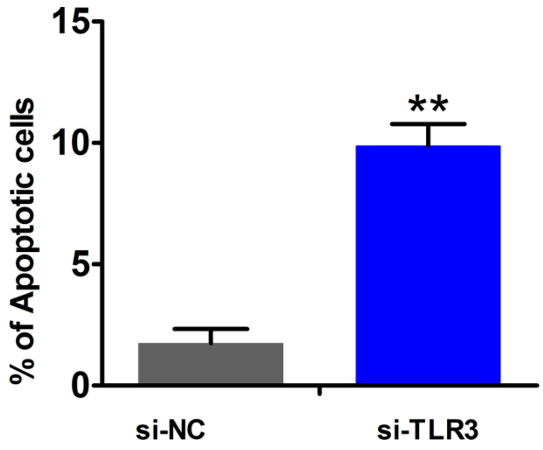

Supplementary Figure 3. When TLR3 knockdown, apoptosis of HepG2 cells was suppressed compared with the control group. ** *P*-value< 0.01.
