# Supplementary material for: In situ self-assembly of Au-antimiR-155 nanocomplexes mediates TLR3-dependent apoptosis in hepatocellular carcinoma cells
Source: Aging (Albany NY). 2020 Nov 5;13(1):241–61. doi: 10.18632/aging.103799 (PMC7834998; doi:10.18632/aging.103799)
Supplement: Supplementary Tables [file aging-13-103799-s002.pdf]

SUPPLEMENTARY TABLES

Supplementary Table 1. General clinical characteristics of 30 HCC tissues.

Characteristics	HCC Patients (n=30)
Males n %	24(80)
Female n %	6(20)
Age (years)	69.03 ± 8.91
PT(s)	14.3±2.7
ALB(g/L)	37.41±7.28
TBIL(μmol/L)	25.72±10.34
ALT (IU/L)	76.25± 41.03
AST (IU/L)	80.27 ± 40.32
HbsAg (Yes/No)	25(83.3)/5(16.7)
HBV-DNA $\log_{10}$ cop/mL	6.39 ± 0.72
AFP(IU/mL)	476± 340.6
TNM stage	
I	7(23.3)
II	11(36.7)
III	8(26.7)
IV	4(13.3)
BCLC stage	
A	17(56.7)
B	8(26.7)
C	4(13.3)
D	1(3.3)
Smoking n %	18(60)
Alcoholism n %	24(80)

[#] Measurement data are mean± standard deviation (SD).

[##] PT, prothrombin time; ALB, albumin; TBIL, total bilirubin; ALT, alamine aminotransferase; AST, aspartate transaminase; HbsAg, hepatitis B surface antigen; HBV, hepatitis B virus; AFP, alpha-fetoprotein; BCLC, Barcelona Clinic Liver Cancer; TNM, tumour, node and metastasis.

Supplementary Table 2. mRNA and miRNA primer sequences.

Primers	Sequences 5' → 3'
miR-155	F primer GCGAAAGCATTTGCCAAGAA<br>R primer CATCACAGACCTGTTATTGC
U6	F primer CAGCACATATACTAAAATTGGAACG<br>R primer ACGAATTTGCGTGTCATCC
TLR3	F primer CCTGGTTTGTTAATTGGATTAACGA<br>R primer TGAGGTGGAGTGTTGCAAAGG
NF-κB (p65)	F primer ATCCCATCTTTGACAATCGTGC<br>R primer CTGGTCCCGTGAAATACACCTC
caspase-8	F primer CATCCAGTCACTTTGCCAGA<br>R primer GCATCTGTTTCCCCATGTTT
GAPDH	F primer TCTCTGCTCCTCCTGTTCGA<br>R primer GCGCCCAATACGACCAAATC
Mimics miR-155	CUCCUACAUAUUAGCAUUAACA<br>GAGGAUGUAUAAUCGUAAUUGU
Mimic-NC	UUCUCCGAACGUGUCACGUUT<br>ACUUGACACGUUCGGAGAATT
Inhibitors-miR-155	UGUUAAUGCUAAUAUGUAGGAG
Inhibitor-NC:	CAGUACUUUUGUGUAGUACAA

miR-155, microRNA-637; U6, U6 small nuclear RNA; TLR3, Toll-like receptor 3; NF-κB, nuclear factor-k-gene binding; caspase-8, cysteinyl aspartate specific proteinase 8; GAPDH, glyceraldehyde-3-phosphate dehydrogenase.

Supplementary Table 3. Sequences of three siRNA of TLR3.

Name	Sequences (5'-3')
siTLR3-1	GAAGCTATGTTTGGAATTA
siTLR3-2	GGAGCACCTTAACATGGAA
siTLR3-3	ATAGGTGCCTTTCGTCATA

Supplementary Table 4. The antibodies employed for the Western blot.

Antibody	Working dilutions
TLR3	1:1000
	1:100
NF-κB (p65)	1:1000
	1:100
Caspase-8	1:1000
	1:100
